# Publication Productivity Among Academic Orthopaedic Surgeons in Canada

**DOI:** 10.7759/cureus.8441

**Published:** 2020-06-04

**Authors:** Yousif Atwan, Brynn P Charron, Sahil Sidhu, Joseph Cavanagh, Ryan Degen

**Affiliations:** 1 Division of Orthopaedic Surgery, Western University, London, CAN; 2 Orthopaedics, Schulich School of Medicine and Dentistry, Western University, London, CAN

**Keywords:** h index, bibliometrics, orthopaedic surgery, academic medicine, academic promotion, gender disparity

## Abstract

Objective

The Hirsch Index (h-index) and m-index are often utilized to assess academic productivity and have been widely found to have a positive association with academic promotion and grant selection. The aim of this study was to assess the relationship between these indices and academic ranks among Canadian orthopaedic surgery faculty members.

Methods

Five hundred and sixty-seven Canadian orthopaedic surgery faculty members associated with residency training programs were included in the study. H-indices of individual faculty members were obtained through Elsevier’s Scopus database. Faculty members’ year of residency graduation was recorded from their respective licensing body database and was utilized as a surrogate for the start of their academic career to determine career duration and calculate the m-index. Faculty members were divided based on their academic rank (assistant, associate and full professors) and subspecialty.

Results

Increased h-index, m-index and long career duration were associated with increased academic rank, while gender did not demonstrate an association. Overall, males had a significantly higher h-index compared to females, but no significant difference was observed when comparing the m-index between genders. The m-index varied between subspecialties among senior faculty, but not among junior-ranked faculty.

Conclusion

Bibliometric academic productivity using h-index and m-index is associated with academic ranking among Canadian orthopaedic surgeons at training institutions. Although these indices may provide insight into the academic merits of faculty members, caution must be taken about utilizing it indiscriminately and their limitations must be strongly considered.

## Introduction

Assessing the quality of academic performance has always been an important consideration for funding agencies, grant allocation and committees evaluating faculty recruitment and promotion [1,2]. The utilization of bibliometric indices to assess academic productivity was first popularized by Eugene Garfield in 1955 with the introduction of the Science Citation Index [3]. This innovation subsequently led to his creation of the “Impact Factor”, which was utilized to assess both author and journal impact [4]. For over four decades, an individual’s academic contribution was classified based on their cumulative citation count, total publication count and/or a journal’s impact factor. Due to the variability in the utilization of these numbers by different institutions, there was a tremendous need for the development of a standardized and objective tool to measure academic performance.

To combat this void, Jorge Hirsch introduced his novel bibliometric index, the h-index [5]. He defined it as follows: “A scientist has index h if h of his or her N_p_ papers have at least h citations each and the other (N_p_- h) papers have ≤h citations” [5]. For example, if a scientist has an h-index of 9, this means that they have published nine papers where each has at least nine citations. Hirsch further described the m-index, which takes into account an individual’s academic career duration in relation to their h-index. The m-index is derived by dividing the h-index by career duration. This, therefore, permitted the comparison of scientists within the same field but at different stages of their academic careers [5,6].

Since its conception, the h-index has been widely calculated and popularized by databases such as Thomson Reuters’ Web of Science, Google Scholar and Elsevier’s Scopus [1,7,8]. It has also been studied in various medical specialties such as anesthesiology [9], general surgery [7], neurosurgery [10,11], ophthalmology [12], otolaryngology [13], radiation oncology [14], radiology [15] and urology [16]. These studies have largely demonstrated a positive association between the h-index and academic promotions. Furthermore, it has been shown that the h- and m-indices increase with academic rank among faculty members at academic orthopaedic surgery institutions in the United States [6,17].

Our study aimed to evaluate the academic productivity of clinical and academic orthopaedic surgeons at training programs across Canada through the h-index and m-index. Study participants were further evaluated based on their academic rank and respective subspecialties.

## Materials and methods

To obtain the list of all orthopaedic surgery faculty members, the Canadian Resident Matching Service (CaRMS) was utilized to outline the 17 orthopaedic surgery training programs across Canada. Each program’s website provided a list of faculty members and their clinical or academic ranking. Programs’ websites were accessed between February and March 2020. Provincial licensing websites, such as that of the College of Physicians and Surgeons of Ontario (CPSO), were utilized to obtain the year of graduation of each faculty member from their respective training programs. This was used as a surrogate for the start of their respective academic career. This value was subtracted from the current year (2020) to determine their academic career duration in years.

Elsevier’s Scopus database (www.scopus.com) was accessed through our institution’s library subscription. A custom search utilizing the name search field was completed and the h-index for each faculty member was recorded. The overall cohort of orthopaedic surgeons was subdivided into clinical and academic surgeons based on their department/university website listing. Those with the wording of adjunct, affiliate, clinical and lecturer were also included within the clinical category. Faculty members hired under academic ranks were divided based on their ranks. Junior academic faculty members included assistant professors, while senior academic faculty members included associate and full professors. This complemented the ranking methodology used in a similar analysis of orthopaedic surgeons in the United States [17]. Furthermore, academic faculty members’ subspecialties were recorded. Exclusion criteria included faculty members without physician degrees, those retired from orthopaedic surgery practice and those with an expired or inactive license. One university was excluded as an updated list of their faculty was not available.

All analyses were completed using IBM SPSS Statistics software (IBM, Armonk, NY). The median and interquartile range (IQR) were calculated for the h-index, career duration and m-index of all faculty members. These metrics were compared between faculty type (academic vs. clinical), academic rank (junior faculty vs. senior faculty), gender (female vs. male) and subspecialty (divided into arthroplasty, foot and ankle, oncology, pediatrics, spine, sports, trauma and upper extremity). Discrete variables were compared using the chi-square test. Non-normally distributed data were compared using the Wilcoxon-Mann-Whitney test for two-group analysis and the Kruskal-Wallis test for analysis of multiple groups. Multivariate regression analysis was completed using nominal logistic regression to assess h-index, career duration and m-index. A p-value of <0.05 was considered statistically significant.

## Results

Faculty rank and distribution

A total of 567 Canadian orthopaedic surgery faculty members were included in the analysis. Of these, 14.5% were clinical faculty (n = 82) and 85.5% were academic faculty (n = 485). Among academic faculty, 58% were assistant professors (n = 280) and were designated as junior faculty. The remaining 42% (n = 205) included 132 associate professors and 73 full professors and were classified as senior faculty.

Clinical and academic faculty

Academic faculty had significantly higher publication productivity metrics when compared with clinical faculty. The median h-index was 8 (IQR: 3 - 16.5) for academic faculty compared to a median of 2 (IQR: 1 - 4) for clinical faculty (p: <0.001). The median career duration was four years longer for academic faculty compared with clinical faculty, with 19 years (IQR: 12 - 28 years) for academic faculty compared with 15 years (IQR: 9-24 years) for clinical faculty (p = 0.031). The median m-indices were 0.50 (IQR: 0.19 - 0.92) and 0.17 (IQR: 0.03 - 0.40) for academic faculty and clinical faculty respectively (p: <0.001).

Academic orthopaedic surgeons

The median h-indices were 4 (IQR: 2 - 9) for assistant professors, 12 (IQR: 6 - 18) for associate professors, and 28 (IQR: 19 - 40) for full professors (p: <0.001). The median career duration was 10 years (IQR: 8 - 21 years) for assistant professors, 23 years (IQR: 17 - 30 years) for associate professors, and 30 years (IQR: 24 - 36 years) for full professors (p: <0.001). The median m-indices were 0.33 (IQR: 0.10 - 0.73) for assistant professors, 0.60 (IQR: 0.25 - 0.89) for associate professors and 0.96 (IQR: 0.53 - 1.52) for full professors (p: <0.001). Productivity metrics for juniors compared with senior academic faculty can be found in Table 1. A higher h-index, m-index and longer career duration demonstrated an independent association with senior academic rank. Meanwhile, gender did not demonstrate such an association (Table 2).

**Table 1:** Overall characteristics of Canadian orthopaedic surgeons *Statistically significant with a p-value of <0.05 IQR: interquartile range

Characteristics	H-index, median (IQR)	P-value	Career duration, years, median (IQR)	P-value	M-index, median (IQR)	P-value
All faculty		<0.001*		0.031*		<0.001*
All clinical orthopaedic surgeons (n = 82)	2 (1 - 4)		14.5 (9 - 24)		0.17 (0.03 - 0.40)	
All academic orthopaedic surgeons (n = 485)	8 (3 - 16.5)		19 (12 - 28)		0.50 (0.19 - 0.92)	
Gender		<0.001*		<0.001*		0.052
All males (n = 430)	8 (3 - 17)		20 (12 - 29)		0.5 (0.20 - 0.94)	
All females (n = 55)	5 (2 - 8)		15 (8 - 22)		0.38 (0.13 - 0.67)	
Academic rank		<0.001*		<0.001*		<0.001*
All junior faculty (n = 280)	4 (2 - 8.75)		10 (8 - 21)		0.33 (0.10 - 0.73)	
All senior faculty (n = 205)	15 (9 - 26)		26 (19 - 33)		0.67 (0.37 - 1.04)	
Junior faculty by gender		0.049*		0.127		0.394
Male (n = 242)	4 (2 - 9)		15 (8 - 21.25)		0.33 (0.11 - 0.75)	
Female (n = 38)	2.5 (1 - 6)		11 (7 - 22)		0.33 (0.07 - 0.56)	
Senior faculty by gender		0.001*		<0.001		0.191
Male (n = 188)	17 (9 - 27.75)		27 (20 - 33)		0.69 (0.37 - 1.04)	
Female (n = 17)	9 (5.50 - 11)		17 (14 - 22)		0.53 (0.21 - 0.79)	

**Table 2:** Multivariate analysis of factors associated with senior academic rank *Statistically significant with a p-value of <0.05 CI: confidence interval

Variable	Odds ratio (95% CI)	P-value
H-index	1.14 (1.11 - 1.17)	<0.001*
Career duration	1.11 (1.09 - 1.14)	<0.001*
M-index	1.76 (1.31 - 2.37)	<0.001*
Female gender	0.58 (0.32 - 1.05)	0.073

Differences between subspecialties

Of the 485 academic orthopaedic surgeons, 455 had specified subspecialties listed. Of these subspecialists, 53 (11.6%) were female. Among these subspecialists, arthroplasty was the most common subspecialty among males (24.4%) while pediatrics was the most common among females (34%). Meanwhile, oncology represented the least common specialty among both females (1.9%) and males (3.7%). The subspecialty with the largest proportion of females was pediatrics (31.6%), while spine had the least (3.3%). Between all subspecialists, there was a statistically significant difference in the h-index (p = 0.004) and m-index (p = 0.001), but not in career duration (Table 3). Upon analysis of junior-ranked subspecialists, there was a statistically significant difference in h-index (p = 0.048) but no difference within career duration (p = 0.840) or m-index (p = 0.125) among the various subspecialties. However, there were statistically significant differences in h-index (p = 0.013) and m-index (p = 0.011) between subspecialties among senior faculty members.

**Table 3:** Overall characteristics of Canadian academic orthopaedic surgeons divided by subspecialty *Statistically significant with a p-value of <0.05 IQR: interquartile range

Characteristics	Number	H-index, median (IQR)	P-value	Career duration, years, median (IQR)	P-value	M-index, median (IQR)	P-value
All academic specialists	455		0.004*		0.705		0.001*
Arthroplasty	106	9 (3 - 19.25)		21 (12 - 30)		0.52 (0.23 - 1.19)	
Foot and ankle	28	3.5 (2 - 7.5)		21 (10 - 29)		0.26 (0.07 - 0.58)	
Oncology	16	10 (6 - 25.25)		20 (12 - 28)		0.57 (0.24 - 1.30)	
Pediatrics	57	5 (2.5 - 9.5)		20 (11 - 30)		0.30 (0.15 - 0.66)	
Spine	61	11 (4.5 - 18.5)		21 (10 - 31)		0.57 (0.25 - 1.07)	
Sports	72	9 (4 - 17.5)		19 (12 - 28)		0.53 (0.20 - 0.93)	
Trauma	61	10 (5.25 - 18.75)		18 (14 - 26)		0.71 (0.26 - 1.04)	
Upper extremity	67	9 (4 - 18)		17 (11 - 24)		0.68 (0.36 - 0.94)	
Junior faculty by subspecialty	251		0.048*		0.840		0.125
Arthroplasty	57	4 (2 - 10)		14 (7 - 23)		0.43 (0.13 - 0.93)	
Foot and ankle	19	3 (1 - 6)		15 (8 - 23)		0.30 (0.07 - 0.50)	
Oncology	9	6 (2.50 - 10)		13 (10 - 20)		0.32 (0.22 - 0.93)	
Pediatrics	28	3.5 (1.25 - 5)		11 (7 - 21)		0.23 (0.08 - 0.55)	
Spine	30	5.5 (2 - 11)		15 (8 - 26)		0.32 (0.12 - 0.89)	
Sports	41	6 (2 - 12)		16 (7 - 22)		0.36 (0.12 - 1.06)	
Trauma	25	6 (1 - 11)		16 (13 - 22)		0.40 (0.05 - 1.00)	
Upper extremity	42	6 (3 -10)		14 (9 - 20)		0.51 (0.31 - 0.80)	
Senior faculty by subspecialty	204		0.013*		0.455		0.011*
Arthroplasty	49	18 (9 - 33.5)		27 (22 - 34)		0.62 (0.33 - 1.41)	
Foot and ankle	9	5 (2.5 - 22)		28 (22 - 34)		0.23 (0.08 - 0.93)	
Oncology	7	26 (13 - 34)		29 (21 - 35)		0.91 (0.51 - 1.62)	
Pediatrics	29	9 (7 - 17)		25 (20 - 34)		0.43 (0.21 - 0.73)	
Spine	31	17 (11 - 26)		26 (20 - 32)		0.67 (0.43 - 1.24)	
Sports	31	12 (8 - 26)		24 (17 - 32)		0.67 (0.48 - 0.93)	
Trauma	23	18 (10 - 31)		24 (15 - 30)		0.86 (0.63 - 1.13)	
Upper extremity	25	19 (12 - 26.50)		24 (17 - 33)		0.76 (0.55 - 0.96)	

Gender and academic rank 

Gender data were analyzed for 567 surgeons, of whom 71 (12.5%) were female. Fifty-five females were academic faculty (77.5% of all female faculty) compared with 430 males (86.7% of all male faculty) (p = 0.039). Among academic faculty, the proportion of females in senior positions was 30.9% (n = 17) compared to 43.7% (n = 188) of males in these positions (p = 0.07). Females represented 13.6% of the assistant professors, 11.4% of the associate professors, and 2.4% of the full professors. Female academic surgeons had a significantly lower median h-index of 5 compared with male academic surgeons (median h-index of 8) (p: <0.001); however, there were no significant differences in the m-index between males and females overall (p = 0.052), male and female junior faculty (p = 0.394) or male and female senior faculty (p = 0.191) (Table 1).

## Discussion

In the last decade, the h-index has become increasingly recognized as a powerful metric of an individual’s academic productivity [18,19]. Prior to its inception, the total number of publications and/or citations was utilized to assess a faculty member’s productivity within their respective field. As a single numeric measure, the h-index provides a more complete overview of the authors’ research impact, and institutions have subsequently adopted it for determining superiority among applicants for awards, grants, promotions and for hiring purposes [2,10,20]. This study assessed academic productivity using this metric and its variants among Canadian orthopaedic surgeons. Increased h-index, m-index and career duration were associated with increased academic rank, while gender did not demonstrate an association.

Similar to the findings of Ence et al. in the United States, we found that the h-index differs significantly among clinical and academic orthopaedic surgery faculty at Canadian training programs. Canadian academic faculty had slightly higher h-index median (8, IQR: 3 - 16.5) and m-index median (0.50, IQR: 0.20 - 0.94) compared to their colleagues in the United States (5, IQR: 1 - 12 and 0.37, IQR: 0.13 - 0.60 respectively) [17]. This study also demonstrated a significant correlation between faculty rank and their respective h-index, career duration and m-index, which is consistent with findings in various other medical and surgical specialties [6,10-13,16]. This correlation suggests that these academic achievement metrics have had a role in the advancement of academic ranks among Canadian orthopaedic surgeons. Furthermore, among academic orthopaedic surgeons, gender was not an independent predictor for their academic rank.

To our knowledge, this is the first study to analyze the academic productivity of various orthopaedic subspecialties. Statistical significance among the subspecialties was noted in junior and senior ranks. Pediatrics and foot and ankle tended to have lower h-indices and this is in line with the findings of the previous studies that subspecialties with lower industry support tended to have lower academic productivity [21,22]. Forrester et al. suggested that the median industry compensation of male surgeons was higher than that of their female colleagues [21]. Their analysis also exhibited disparity among orthopaedic subspecialties. Lieber et al. further demonstrated an association between increased industry consulting payments and academic productivity among orthopaedic surgeons in the United States [22].

Gender disparities in medical and surgical specialties have been extensively studied over the past few decades. These studies have demonstrated an underrepresentation of females across a number of medical and surgical specialties, including orthopaedic surgery [23]. Although Martinez et al. demonstrated no differences in h-index between female and male musculoskeletal oncology surgeons, our study demonstrated higher h-indices for male orthopaedic surgeons compared to their female colleagues. This difference, along with the shorter career duration among females, is also consistent with the demographics of orthopaedic surgeons in the United States [17]. Nonetheless, it is important to note that this h-index disparity is not evident when examining the m-index within the same cohort. It has been previously hypothesized that female academic productivity based on the h-index is lower compared to their male colleagues at the early stages of their careers, due to the period coinciding with child-bearing years, and it subsequently balances out in senior years [24]. Our study suggests that this disparity that is balanced by the m-index may in fact be due to an age disparity as the m-index takes into account their academic duration. The Canadian Medical Association’s profile of Canadian orthopaedic surgeons in 2018 determined that 62.6% of females were under the age of 45 while only 32.3% of their male colleagues were within that age group [25]. This likely accounts for the disparity that is only seen when comparing h-indices and not m-indices. The significant career duration difference may also be attributed to a younger female cohort, rather than an overall shorter career, as a number of studies have demonstrated an increased representation of females in orthopaedic surgery in recent years [23,26].

Despite the wide utilization of the h-index as a marker for academic productivity, its limitations should be thoroughly considered. Since its inception, Hirsch has discouraged its use in comparing individuals of different specialties as the number of readers and researchers varies between specialties [5]. Also, Svider et al. demonstrated differences in h-indices among academic surgical specialties [27]. This phenomenon could account for the variance noted in our study among orthopaedic surgery subspecialties. Although they are all within the same “specialty”, certain subspecialties may include readership among other disciplines. For example, a medical oncology journal would be more likely to host orthopaedic oncology publications in comparison to foot and ankle studies. This should also caution surgical departments about utilizing generalized h-index cut-offs for promotion or other evaluative purposes between all surgical specialties or subspecialties. Also, the h-index does not compensate for confounders such as the “Matthew effect” whereby more notable researchers may be cited at an increased rate in comparison to their less established colleagues [28]. It has also been suggested that the practice of “gratuitous authorship” can confound calculated h-indices as each co-author receives equal credit when utilizing this metric. Lee et al. disputed this notion as their study of academic neurosurgical faculty did not yield a change in h-index at the individual level when assessing the position of authorship [10]. Lastly, the muddling of self-citation has also been a criticism of the h-index [29]. Engqvist et al. examined this issue and found a minimal effect on the h-index after removing self-citations from the authors studied [29].

Aside from the known limitations of the indices utilized, this study also has a number of other limitations. The utilization of the database, Elsevier’s Scopus, to determine an individual’s h-index has the potential to credit a publication to the wrong author and we would not be able to correct that. Nonetheless, studies have demonstrated no significant difference in calculated h-index when comparing Google Scholar and Elsevier’s Scopus and therefore its use as the only source was justified [6]. As for other limitations, the analysis of this database may not take into account faculty who have changed their names in the midst of their careers. This may potentially disadvantage female faculty who change their name in relation to marital status. Nonetheless, a study assessing the academic productivity of radiation oncologists noted that 12.4% of their female faculty had published using more than one name during their academic career and this had affected the h-index in approximately 8.2% of the cases [30].

## Conclusions

Overall, the difference in h-indices between male and females, and junior and senior faculty among Canadian orthopaedic surgeons was similar to their colleagues in the United States. After adjusting for career duration by utilizing the m-index, the data for both countries did not demonstrate differences in terms of gender. Although these indices correlate with academic advancement, caution should be taken in their utilization for the establishment of advancement criteria as they are prone to bias, confounding and do not necessarily represent the full academic potential of an individual.
